# A cell-based high-throughput screen identifies inhibitors that overcome P-glycoprotein (Pgp)-mediated multidrug resistance

**DOI:** 10.1371/journal.pone.0233993

**Published:** 2020-06-02

**Authors:** Rida Zahra, Muhammad Furqan, Rahim Ullah, Aziz Mithani, Rahman Shah Zaib Saleem, Amir Faisal

**Affiliations:** 1 Department of Biology, Syed Babar Ali School of Science and Engineering, Lahore University of Management Sciences, Lahore, Pakistan; 2 Department of Chemistry & Chemical Engineering, Syed Babar Ali School of Science and Engineering, Lahore University of Management Sciences, Lahore, Pakistan; Columbia University, UNITED STATES

## Abstract

Multidrug resistance (MDR) to chemotherapeutic drugs remains one of the major impediments to the treatment of cancer. Discovery and development of drugs that can prevent and reverse the acquisition of multidrug resistance constitute a foremost challenge in cancer therapeutics. In this work, we screened a library of 1,127 compounds with known targets for their ability to overcome Pgp-mediated multidrug resistance in cancer cell lines. We identified four compounds (CHIR-124, Elesclomol, Tyrphostin-9 and Brefeldin A) that inhibited the growth of two pairs of parental and Pgp-overexpressing multidrug-resistant cell lines with similar potency irrespective of their Pgp status. Mechanistically, CHIR-124 (a potent inhibitor of Chk1 kinase) inhibited Pgp activity in both multidrug-resistant cell lines (KB-V1 and A2780-Pac-Res) as determined through cell-based Pgp-efflux assays. Other three inhibitors on the contrary, were effective in Pgp-overexpressing resistant cells without increasing the cellular accumulation of a Pgp substrate, indicating that they overcome resistance by avoiding efflux through Pgp. None of these compounds modulated the expression of Pgp in resistant cell lines. PIK-75, a PI3 Kinase inhibitor, was also determined to inhibit Pgp activity, despite being equally potent in only one of the two pairs of resistant and parental cell lines. Strong binding of both CHIR-124 and PIK-75 to Pgp was predicted through docking studies and both compounds inhibited Pgp in a biochemical assay. The inhibition of Pgp causes accumulation of these compounds in the cells where they can modulate the function of their target proteins and thereby inhibit cell proliferation. In conclusion, we have identified compounds with various cellular targets that overcome multidrug resistance in Pgp-overexpressing cell lines through mechanisms that include Pgp inhibition and efflux evasion. These compounds, therefore, can avoid challenges associated with the co-administration of Pgp inhibitors with chemotherapeutic or targeted drugs such as additive toxicities and differing pharmacokinetic properties.

## Introduction

Chemotherapy is one of the most common and successful treatments for cancers. The success of chemotherapy, however, can be limited by several factors including toxicity and the development of multidrug resistance (MDR), where cancer cells become resistant to structurally and functionally unrelated drugs[1]. MDR is one of the major mechanisms through which tumors acquire drug resistance and is thereby responsible for the failure of many chemotherapeutic and targeted drugs[2]. Among various mechanisms that contribute to the development of multidrug resistance, overexpression of ABC (ATP binding cassette) transporters is the most prevalent. Forty-eight ABC transporters from seven different families (ABCA-ABCG) have been identified in the human genome, and nearly twenty of them act as drug efflux pumps. These include Permeability glycoprotein (Pgp), MDR-associated protein 1 (MRP1) and breast cancer resistant protein (BCRP), among others[3]. *MDR1-*encoded Pgp consists of two transmembrane domains and two nucleotide-binding domains. Cells overexpressing *MDR1* are resistant to a wide range of drugs such as anthracyclines, taxanes, vinca alkaloids, epipodophyllotoxins, actinomycin D and colchicines, which are all efflux substrates of Pgp[2, 4]. *MDR1* overexpression can result from gene amplification or transcriptional activation[5, 6].

Multidrug resistance in Pgp overexpressing cells can be overcome by inhibition of Pgp expression, interference with its activity or by avoiding the efflux through it(2). Perifosine and dasatinib, for example, downregulate Pgp expression by inhibiting the Akt/PI3K/NF-kB[7] and the Erk[8] pathways, respectively. Similarly, ZSTK474 inhibits the expression of two ABC transporters, Pgp and MRP1[9]. Ceritinib (LDK378) on the other hand, sensitizes ABCB1 and ABCG2 overexpressing cell lines to conventional drugs through a mechanism that involves competitive inhibition of ABCB1 and ABCG2[10]. Likewise, saquinavir (an HIV protease inhibitor), itraconazole and ketoconazole (Azole antifungals) also competitively inhibit the transport function of Pgp[11, 12]. Propafenone, progesterone, gomisin A, valspodar and elacridar are examples of non-competitive Pgp inhibitors that bind to an allosteric site of Pgp[13]. Some drugs such as epothilone B, annamycin, and MPC 6827 can escape the efflux as they are not substrates of Pgp[2, 14–16].

Several compounds with the ability to reverse Pgp-mediated multidrug resistance have been evaluated in the clinic without much success[17]. This is mainly due to the associated toxicities at the concentrations required for effective inhibition of the efflux pumps[18]. Verapamil, a first-generation inhibitor, for example, is a substrate and a competitive inhibitor of Pgp that failed in clinical trials due to cardiotoxicity[19]. Similarly, a second-generation inhibitor, PSC-833 was also unsuccessful in clinical trials due to altered pharmacokinetic interactions which resulted in the decreased clearance and increased plasma concentration of the inhibitor[20]. Both these inhibitors act as modulators, i.e. they compete with conventional chemotherapeutic drugs at the substrate-binding site of the protein, which results in the increased accumulation of cytostatic drugs within the cell. Tariquidar (XR-9576), a Pgp ATPase inhibitor, showed limited clinical activity in phase II and exhibited unfavorable toxicities in the terminated phase III clinical trial[13, 21]. There is, therefore, a need to identify drugs that can overcome multidrug resistance by either inhibiting the Pgp activity or by avoiding the Pgp-mediated efflux.

High throughput screening of chemical libraries is one of the most common approaches used to identify such drugs, and several Pgp inhibitors have been identified through the cell-based compound library or *in silico* screening approaches[22–25]. Some of these Pgp inhibitors can only sensitize Pgp-expressing cells to chemotherapeutic agents[23] while others have primary activity against cellular targets and therefore, can overcome MDR on their own[24]. In this study, we screened a library of 1,127 inhibitors with known targets in a pair of parental and multidrug-resistant cell lines for their ability to overcome Pgp-mediated multidrug resistance in a 3-day proliferation assay. We identified four inhibitors that were equally potent against two pairs of parental and MDR1 overexpressing cell lines. We also determined the mechanism(s) through which they overcame MDR using cell-based efflux assays. Our results demonstrate that the screening of compound libraries with known cellular targets can identify potent small molecule inhibitors that overcome MDR on their own by inhibiting Pgp or by avoiding efflux through it.

## Materials and methods

### Cell culture

The parental and resistant cell line pairs, KB-3-1/KB-V1, and A2780/A2780-Pac-Res were kindly provided by Professor Michael Gottesman (Centre for Cancer Research, NCI) and Professor Spiros Linardopoulos (Institute of Cancer Research, UK), respectively. All the cell lines were maintained in their respective culture media (DMEM for KB-3-1/KB-V1 and RPMI for A2780/A2780-Pac-Res) supplemented with 10% Fetal bovine serum (FBS) and 1% Anti-anti (Antibiotic and Antimycotic). Cells were cultured at 37°C in humidified incubators with 5% CO_2_ and passaged for less than 6 months before replacement with an earlier frozen stock.

### Primary and secondary screening

Primary screening was carried out with Selleckchem inhibitor library (1,127 compounds procured from Selleck Chemicals, USA) in parental KB-3-1 and *MDR1* overexpressing drug-resistant KB-V1 cell lines using a 3-day Sulforhodamine B (SRB) proliferation assay. Cells were seeded in 96 well plates at their respective seeding densities optimized to yield similar SRB reading at the end of the assay (KB-3-1 = 1500 cells per well and KB-V1 = 3500 cells per well). Twenty-four hours after seeding, cells were treated with the inhibitors at 1 μM concentration or DMSO as vehicle control (0.5%) for 72 hours. At the end of the treatment, the SRB assay was performed as described previously[26]. Briefly, cells were fixed with 10% ice-cold TCA (3% final concentration) for at least 2 hours at 4°C. Following fixation, plates were washed with water, air-dried and incubated with 0.06% SRB (100μl) for 30 minutes. Plates were washed again with 1% acetic acid solution and air-dried in an incubator at 37°C. SRB from the stained cells was dissolved in 100 μl of 10 mM Tris (pH = 10.5) on a shaker, and optical density was measured at 490 nm on a BioTek microplate reader.

For secondary screening, 118 selected inhibitors were screened at a 1 μM concentration in both KB-3-1/KB-V1 and A2780/A2780-Pac-Res cell lines pairs using a 3-day SRB proliferation assay as described above.

### GI_50_ of selected inhibitors

GI_50_ values of 15 inhibitors selected from the secondary screening based on the equal inhibition of cell proliferation in both parental and resistant cell line pairs were determined using SRB proliferation assay as described above. Cells were treated with five three-fold dilutions of the inhibitors for 3 days, and GI_50_ values were calculated using GraphPad Prism.

### Western blotting

Antibodies against alpha-tubulin (sc-8035) and MDR1 (sc-55510) were purchased from Santa Cruz, USA, whereas Horseradish peroxidase (HRP) labeled anti-mouse secondary antibody (1036–05) was purchased from Southern Biotech, USA. For immunoblotting, cells were collected in lysis buffer (100 mM NaCl, 50 mM Tris pH = 7.4, 1% Triton X supplemented with cocktails of phosphatase and protease Inhibitors) on ice, and cell lysates were cleared through centrifugation. Protein concentration was measured by Bradford assay and equal amounts of proteins (15 μg total for each sample) were loaded on 10% SDS-PAGE. Separated proteins were then transferred onto nitrocellulose membrane, followed by blocking with 5% skimmed milk for 1 hour at room temperature. Membranes were then incubated with respective primary antibodies overnight at 4°C. Next day, blots were washed with 1X PBST and incubated with HRP labelled anti-mouse secondary antibody for 1 hour at room temperature. Finally, blots were washed with 1X PBST, developed with enhanced chemiluminescence (ECL) reagent and visualized on BioRad ChemiDoc.

### FACS-based drug efflux assays

FACS-based drug efflux assays were performed using calcein AM and Rhodamine 123 dyes. Cells were trypsinized and pre-treated with the indicated concentrations of the inhibitors (or 0.25% DMSO as vehicle control) for 15 minutes then incubated with 1 μM calcein AM or 5 μM Rhodamine 123 for 1 hour at 37°C. Cells were then pelleted through centrifugation and resuspended in the efflux media (growth media of the cell lines) containing indicated concentrations of the inhibitors and incubated at 37°C for 2 hours. The intracellular accumulation of Rhodamine 123 and calcein (fluorescent hydrolyzed product of calcein AM) was analyzed using BD FACSCalibur.

### Immunofluorescence-based efflux assay

To visualize the intracellular retention of calcein AM, cells were seeded on Poly-L-lysin treated coverslips and pre-treated with the indicated concentrations of the inhibitors (or 0.25% DMSO as vehicle control) for 15 minutes followed by the 1-hour incubation with 1 μM calcein AM at 37°C. After 1 hour, cells were washed twice with PBS and incubated in the efflux media containing indicated concentrations of inhibitors for 2 hours at 37°C. Cells were again washed with PBS and fixed with 4% formaldehyde. Slides were prepared, and images were taken using a Nikon confocal microscope.

### Molecular docking

Molecular docking was performed with the NBD1 of the P-glycoprotein (PDB code: 4Q9H)(27). The structures of CHIR-124 and PIK-75 were drawn using Chem3D. Energy minimization was carried out using Avogadro. The reported 3D structure of P-glycoprotein was retrieved from the Protein Data Bank. This structure was initially calculated using X-ray diffraction with a resolution of 3.4 Å [27]. The addition of hydrogen atoms was performed using MGLTools for AutoDock, and the docking was performed using AutoDock Vina (Scripps Research Institute, USA)[28]. The docking was carried out using exhaustiveness of 8 and grid box dimensions of 50x48x54 encompassing NBD1.

### *In vitro* Pgp-ATPase assay

Pgp-Glo Assay Systems (Promega) was used to determine the effect of CHIR-124 and PIK-75 on the ATPase activity of Pgp. Effect of 2.5 μM concentrations of both the compounds on verapamil-induced ATPase activity of Pgp was determined according to manufacturer’s instructions. Fold change in ATPase activity was calculated, and unpaired t-test was used to determine statistical significance using GraphPad Prism.

## Results

### Identification of compounds that can overcome MDR through high-throughput screening

**Fig 1:**
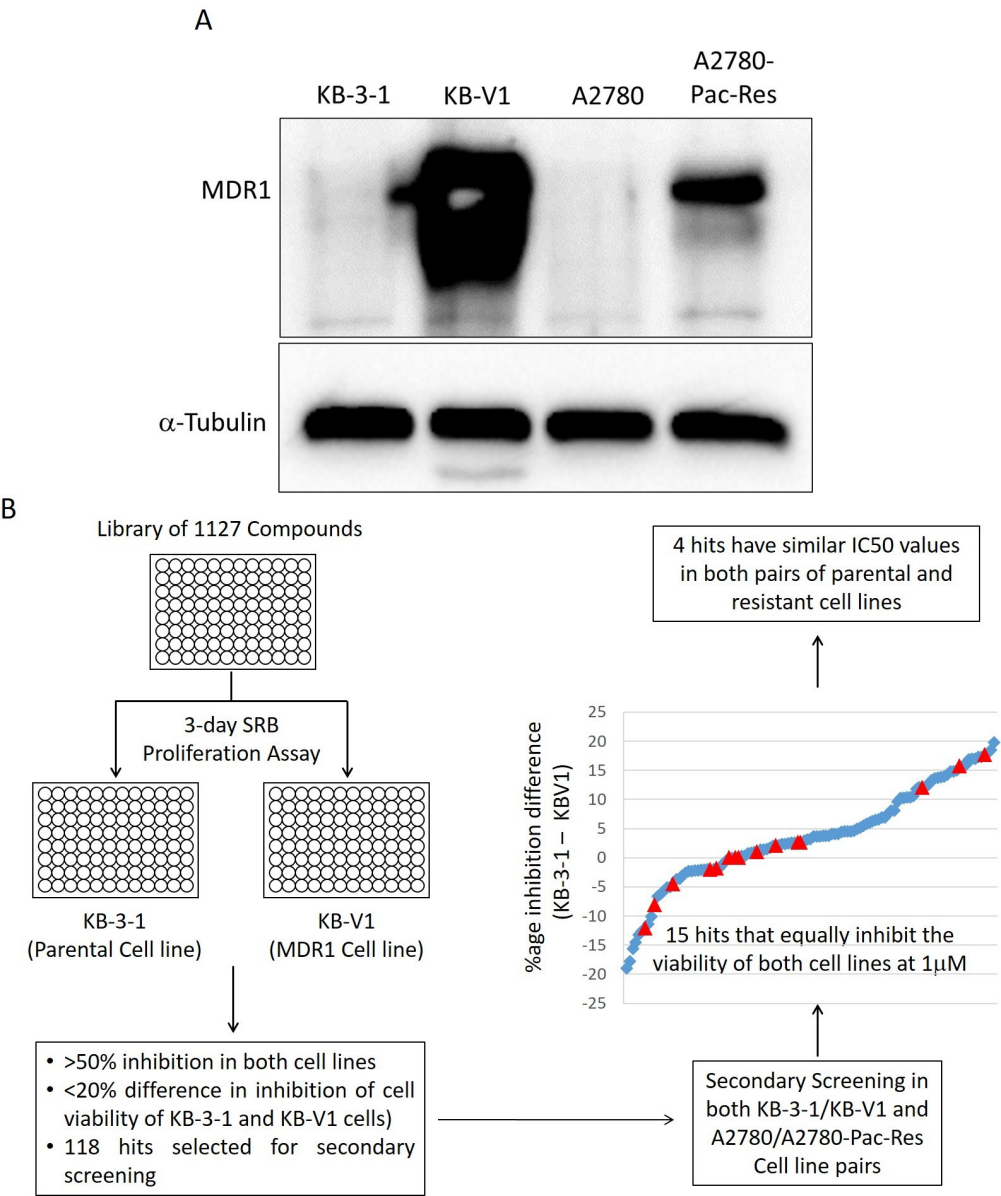
Screening for compounds with the ability to overcome Pgp-mediated MDR. *A*. Expression of Pgp in parental and multidrug-resistance cell line pairs. Both pairs of cell lines were analyzed through western blotting for the expression of Pgp (MDR1). *B*. Flow chart for primary/secondary screening and identification of hits.

In order to identify compounds with the ability to overcome Pgp-mediated MDR, we screened a library of 1,127 inhibitors (Selleckchem inhibitor library) in KB-3-1 cell line, and its multidrug-resistant (Pgp overexpressing) derivative cell line, KB-V1 (Fig 1A). Compounds were screened at 1 μM concentration in a 3-day SRB proliferation assay for their ability to equally inhibit the growth of both parental and Pgp-overexpressing resistant cell lines (Fig 1B). Paclitaxel, a known substrate of Pgp, was used as a control for resistance in the Pgp-overexpressing cell lines. A total of 118 compounds that exhibited more than 50% growth inhibition and less than 20% difference in potency in both the parental and resistant cell lines were selected for further evaluation in the secondary screening (Fig 1B). Most compounds in the primary screening were found to be more potent in the parental KB-3-1 cell line as compared to the multidrug-resistant KB-V1 cell line including some of the known substrates of Pgp (S1 Fig and S1 Table).

### Confirmation of the secondary hits

**Fig 2:**
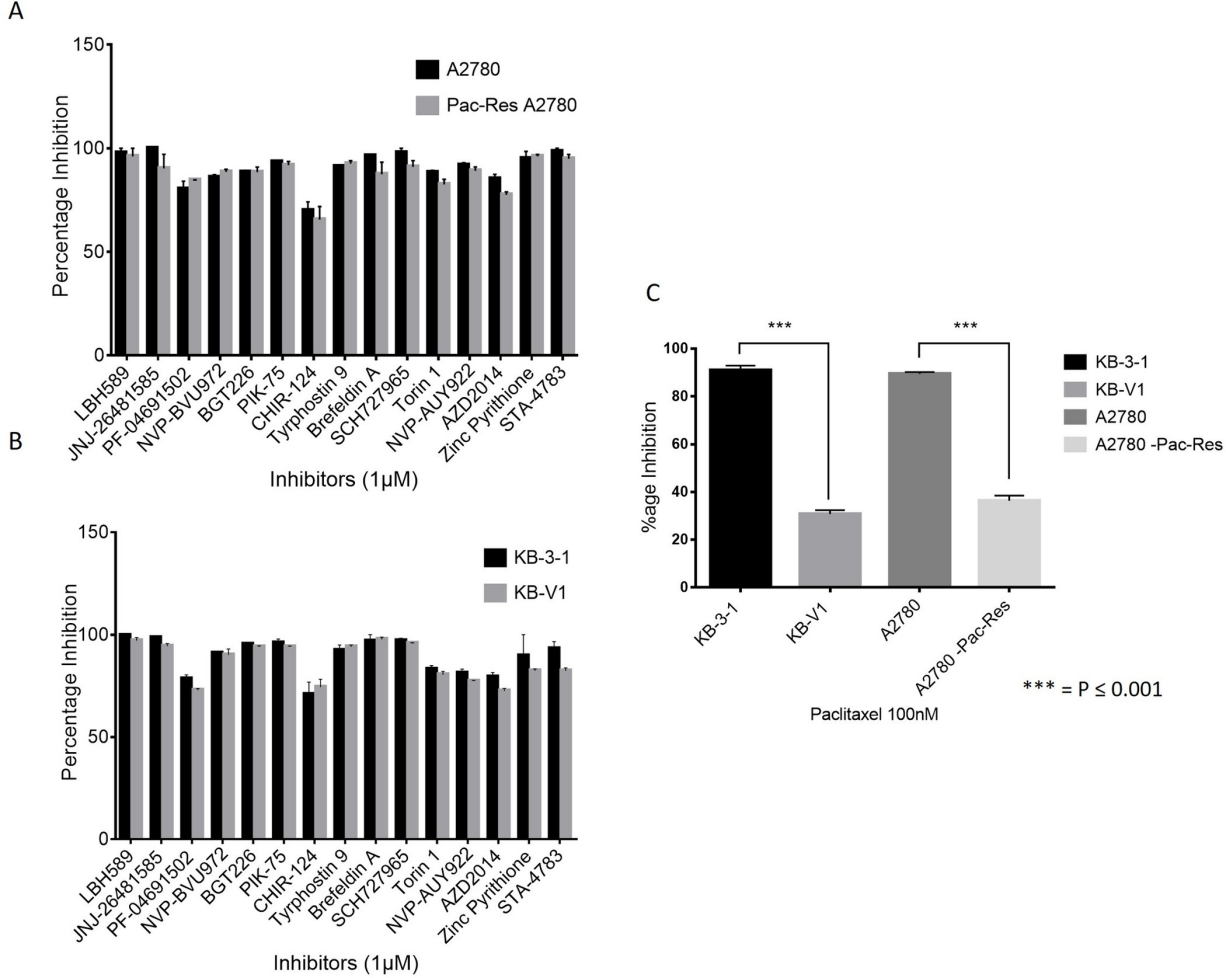
Secondary screening with 118 compounds in both pairs of parental and resistant cell lines. Growth inhibition of A2780/A2780-Pac-Res (*A*.) and KB-3-1 and KB-V1 (*B*.) cell line pairs by 15 hits confirmed in the secondary screening at 1μM concentration. *C*. Growth inhibition of both the cell line pairs by paclitaxel.

The 118 compounds selected from the initial screen were reevaluated through secondary screening for growth inhibition in two pairs of parental and multidrug-resistant cell lines (KB-3-1/KB-V1 and A2780/A2780-Pac-Res)(26) at 1 μM concentration in the 3-day SRB proliferation assay (Fig 1A and 1B). A total of 15 compounds (out of 118) were found to be equally potent against sensitive and resistant cells in both the cell line pairs (KB-3-1/KB-V1 and A2780/A2780-Pac-Res) at 1μM concentration indicating their ability to overcome Pgp-mediated multidrug resistance at this concentration (Fig 2A and 2B). Like the primary screen, paclitaxel was used as a control for resistance in the Pgp-overexpressing cell lines (Fig 2C).

Most of the 15 inhibitors confirmed in the secondary screening inhibited the growth of both the parental and resistant cell lines by approximately 70–100% at 1 μM concentration. At lower concentrations, however, their potency could be different in Pgp-overexpressing resistant cell lines. We, therefore, next determined the half-maximal growth inhibitory concentration (GI_50_) for all the 15 compounds in both these parental and resistant cell line pairs in the 3-day SRB proliferation assay (Table 1). Resistance index (RI; GI_50_ of resistant cell line/ GI_50_ of parental cell line) for all the inhibitors in both cell line pairs were also calculated. Most of the inhibitors had GI_50_ values of less than 1 μM in all four cell lines (Table 1), but only four inhibitors had RI of close to 1 in both pairs of cell lines. CHIR-124 (Chk1inhibitor)[29], tyrphostin-9 (PDGFR inhibitor)[30, 31], brefeldin-A (Protein Transport inhibitor)[32], and elesclomol (Oxidative stress inducer)[33] were equally (or more) potent in KB-V1 compared to KB-3-1 cells with the RI of 1.27, 0.60, 1.32 and 0.69, respectively. Likewise, these four inhibitors also showed similar GI_50_ values in A2780 and A2780-Pac-Res cell line pair with the RI of 0.53, 1.28, 0.88 and 1.08, respectively.

**Table 1:** Resistant Index (RI) of selected inhibitors in parental and resistant cell line pairs.

Inhibitors	GI50 (nM) ± SEM (n = 2)		Resistance Index	GI50 (nM) ± SEM (n = 2)		Resistance Index
	KB-3-1	KB-V1		A2780	A2780-Pac-Res	
Quisinostat (JNJ-26481585)	24.27 ± 10.12	95.07 ± 11.14	3.92	2.94 ± 0.67	207.25 ± 21.65	70.49
Panobinostat (LBH589)	22.51 ± 1.39	55.51 ± 1.88	2.47	28.99 ± 1.83	157.75 ± 24.65	5.44
PF-04691502	127.50 ± 30.01	370.85 ± 35.05	2.91	154.13 ± 85.58	524.95 ± 232.45	3.41
**NVP-BVU972**	1681 ± 246	4770 ± 300	2.84	**2272 ± 178**	**615.85 ± 44.75**	**0.27**
BGT226 (NVP-BGT226)	7.67 ± 2.11	558.35 ± 124.45	72.8	1.16 ± 1.01	158.15 ± 83.86	136.33
**CHIR-124**	**553.65 ± 75.35**	**705.45 ± 77.25**	**1.27**	**417.35 ± 49.05**	**221.85 ± 12.55**	**0.53**
Dinaciclib (SCH727965)	7.88 ± 2.26	24 ± 6.10	3.05	9.13 ± 1.48	75.22 ± 12.15	8.24
**Tyrphostin 9**	**786.80 ± 65.90**	**475.70 ± 123.60**	**0.60**	**340.50 ± 23.70**	**436.55 ± 16.25**	**1.28**
Torin 1	6.1 ± 1.89	317.10 ± 197.40	51.98	0.43 ± 0.19	201.65 ± 99.45	468.95
AZD2014	113.15 ± 8.05	334.65 ± 54.25	2.96	222.90 ± 66.50	611.20 ± 29.60	2.74
**Brefeldin A**	**35.97 ± 7.47**	**47.42 ± 4.91**	**1.32**	**49.59 ± 6.62**	**43.43 ± 5.61**	**0.88**
**Zinc. pyrithione**	163.45 ± 10.85	494.05 ± 51.45	3.02	**89.04 ± 34.46**	**58.69 ± 28.83**	**0.66**
**PIK-75**	**47.44 ± 3.32**	**43.54 ± 2.28**	**0.92**	**41.54 ± 3.11**	**121.65 ± 0.05**	**2.93**
**Elesclomol (STA-4783)**	**12 ± 3.6**	**8.31 ± 0.40**	**0.69**	**3.78 ± 0.20**	**4.08 ± 0.23**	**1.08**
**AUY922 (NVP-AUY922)**	3.18 ± 1.21	13.68 ± 4.95	4.30	**4.32 ± 2.25**	**5.02 ± 1.33**	**1.16**
Paclitaxel	0.66 ± 0.18	480.17 ± 47.1	**728**	2.08 ± 0.63	70.03 ± 10.92	**33.67**

GI_50_ values of all the inhibitors in KB-3-1/KB-V1 and A2780/A2780-Pac-Res cell line pairs were determined (n = 2), and the RI was calculated (GI_50_ of resistant cell line/ GI_50_ of parental cell line).

Three inhibitors, NVP-BVU972 (c-MET inhibitor)[34], zinc pyrithione (a proton pump inhibitor)[35] and AUY922 (HSP90)[36] had RI of close to or less than one (0.27, 0.66 and 1.16 respectively) in A2780/A2780-Pac-Res pair but showed RI of more than 2 (RI of 2.84, 3.02 and 4.30, respectively) in KB-V1/KB-3-1 pair. On the contrary, PIK-75 (PI3K inhibitor)[37] equally inhibited the growth of KB-3-1/KB-V1 pair (RI of 0.92) compared to A2780/A2780-Pac-Res pair (RI of 2.93; Table 1). Both the MDR1 cell lines, KB-V1 and A2780-Pac-Res, were resistant against positive control paclitaxel, compared to their sensitive counterparts (Table 1).

#### CHIR-124 and PIK-75 inhibit Pgp in resistant cells

**Fig 3:**
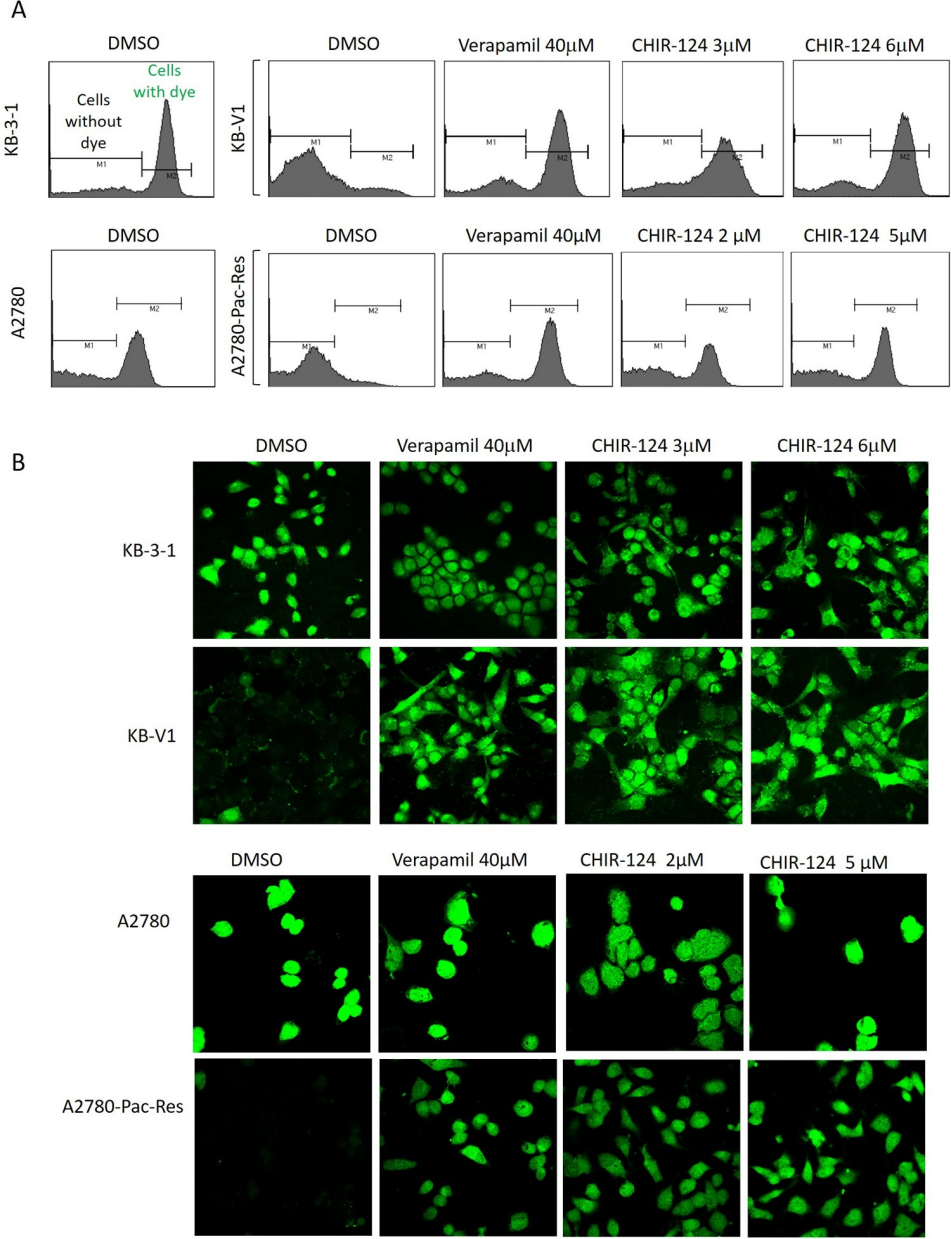
Calcein AM Efflux assays in Pgp-overexpressing multidrug-resistant cell lines. Calcein retention in KB-3-1/KB-V1 and A2780/A2780-Pac-Res parental and resistant cell line pairs. Calcein retention in the presence of DMSO, verapamil and CHIR-124 was determined through FACS analysis (*A*) or immunofluorescence (*B*) as described in the materials and methods.

Following the identification of inhibitors that could overcome MDR, we determined their mechanism of action through cell-based efflux assays. Effect of the inhibitors on the uptake of calcein AM or rhodamine 123, two Pgp substrates, in parental and resistant cell lines was determined through FACS analysis. Majority of the solvent-treated parental KB-3-1 and A2780 cells incubated with calcein AM retained the dye, while very few solvent-treated resistant cells (KB-V1 and A2780-Pac-Res) were positive for green-fluorescent calcein, indicating its efflux from the cells (Fig 3A). Treatment with 40 μM verapamil, a Pgp substrate (that acts as an inhibitor through competition with other substrates), caused a robust increase in calcein positive cells for both the resistant KB-V1 and A2780-Pac-Res cell lines (Fig 3A). Treatment of both the resistant cell lines, KB-V1 and A2780-Pac-Res, with 5x and 10x GI_50_ concentrations of CHIR-124 (in parental cell lines KB-3-1 and A2780), also caused a dose-dependent increase in accumulation of calcein positive cells compared with the solvent-treated controls. Similarly, treatment of A2780-Pac-Res cells with verapamil (40 μM) and CHIR-124 (10 μM) increased the accumulation of Rhodamine-123 in these cells (S2 Fig). Effect of CHIR-124 on calcein retention in resistant cells was also confirmed through immunofluorescence (Fig 3B). CHIR-124 increased the retention of calcein in both KB-V1 and A2780-Pac-Res cells, similar to the one induced by verapamil (Fig 3B).

The PI3-Kinase inhibitor PIK-75 with RI of 0.92 in KB-3-1/KB-V1 pair also inhibited Pgp function in KB-V1 cells as shown by increased calcein retention in both FACS- and immunofluorescence-based assays (S3 Fig). Although the RI of PIK-75 in A2780/A2780-Pac-Res pair was >2 (2.93), the Pgp function in the resistant A2780-Pac-Res cells was also potently inhibited (S3 Fig). Since PIK-75 inhibits Pgp in both the resistant cell lines, the moderate resistance to the inhibitor seen in A2780-Pac-Res cell lines may be independent of Pgp expression in these cells. Two other inhibitors, zinc pyrithione and AUY922, with RI of ~ 1 in only one cell line pair (A2780/A2780-Pac-Res), were also evaluated for inhibition of Pgp in resistant cells (S4 Fig). While both compounds increased calcein retention in A2780-Pac-Res cells (with relatively lower expression of Pgp), there was no increase in calcein retention by either compound in KB-V1 cells (which have relatively higher expression of Pgp). The higher expression of Pgp in these cells could be responsible for lack of calcein retention (following treatment with 1.25 μM and 2.5 μM of AUY922 and zinc pyrithione). This also explains moderate resistance of KB-V1 cells to these inhibitors.

#### Elesclomol, tyrphostin-9 and brefeldin A overcome multidrug resistance by avoiding efflux

**Fig 4:**
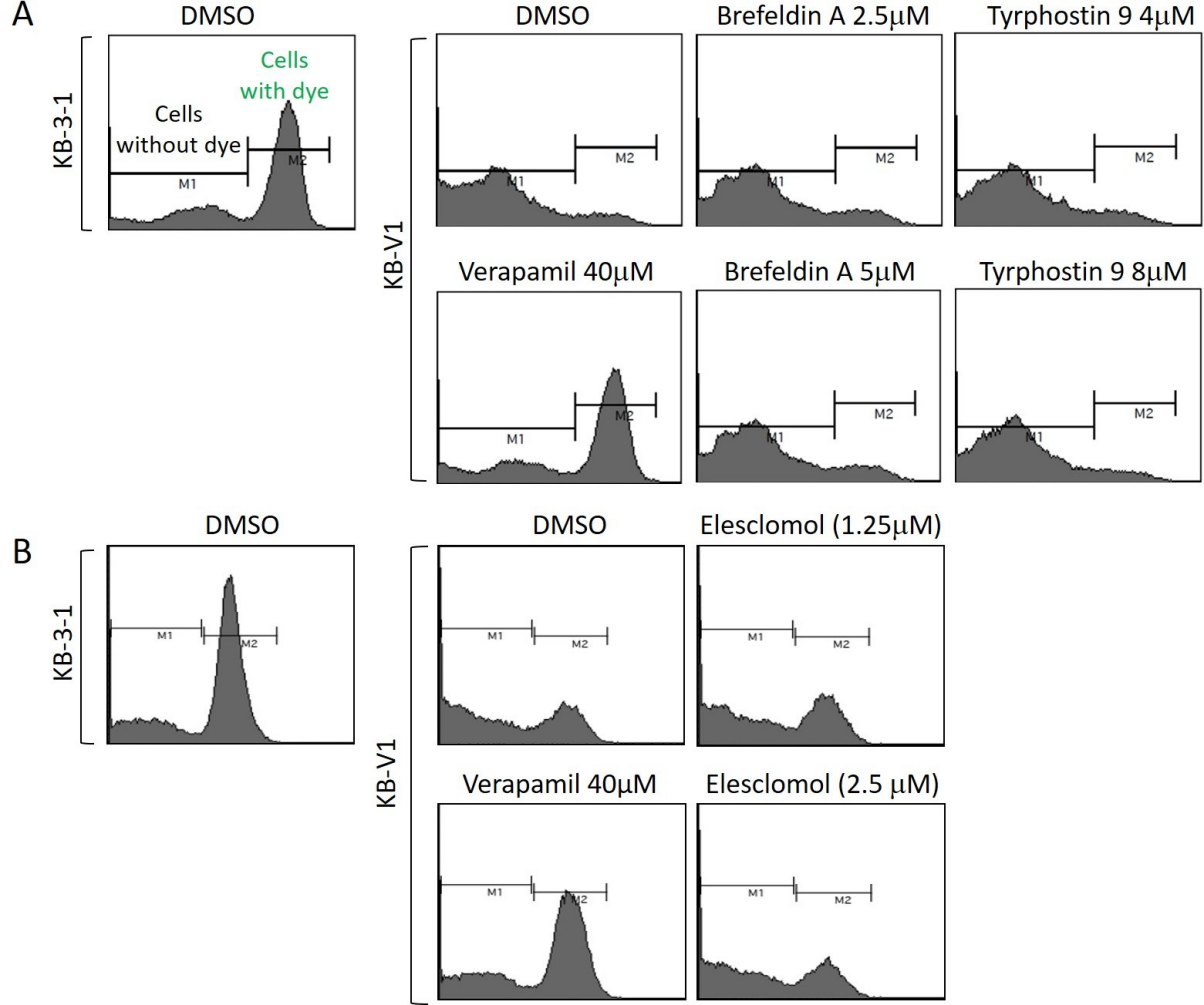
Effect of tyrphostin-9, brefeldin A and elesclomol on Pgp inhibition using calcein AM efflux assay. Calcein retention in parental and resistant cells was determined through FACS analysis following treatment with the indicated concentrations of tyrphostin-9, brefeldin A (*A*) and elesclomol (*B*).

Three other inhibitors, elesclomol, tyrphostin-9 and brefeldin A, were also evaluated in the calcein AM efflux assay as described above. Treatment of the resistant KB-V1 cells with tyrphostin-9 (4 μM and 8 μM), brefeldin A (2.5 μM and 5 μM) or elesclomol (1.25 μM and 2.5 μM) did not result in any change in the efflux of calcein AM compared to the vehicle control, suggesting that there was no accumulation of cells with calcein retention (Fig 4). As expected, control verapamil (40 μM) caused increased retention of calcein AM in the same experiment. Since elesclomol, tyrphostin-9 and brefeldin A inhibit the growth of resistant cell lines with similar potency to parental cell lines (with RI values of 0.69, 0.6 and 1.32 in KB-V1 cells and 1.08, 1.28 and 0.88 in A2780-Pac-Res cells, respectively), their efficacy probably results from them being not substrates of Pgp thereby avoiding the efflux from these cells. Similarly, NVP-BVU972 was unable to inhibit the Pgp function in A2780-Pac-Res cells despite killing the parental and resistant A2780 cell lines with RI of less than 1 indicating that it also overcomes MDR in these cells because it is not a Pgp substrate (S5 Fig).

Downregulation of Pgp expression is another established multidrug resistance reversal mechanism [7, 38–40]. We, therefore, determined whether any of the compounds identified in this screen modulate Pgp expression in resistant cells. Treatment of A2780-Pac-Res cells with these compounds for 24 hours did not significantly alter the expression of Pgp compared to the vehicle controls (S6 Fig). This suggests that these compounds overcome MDR either through inhibition of the Pgp activity or through evasion of efflux by not being Pgp substrates.

### CHIR-124 and PIK-75 are predicted to bind with Pgp and inhibit its ATPase activity *in vitro*

**Fig 5:**
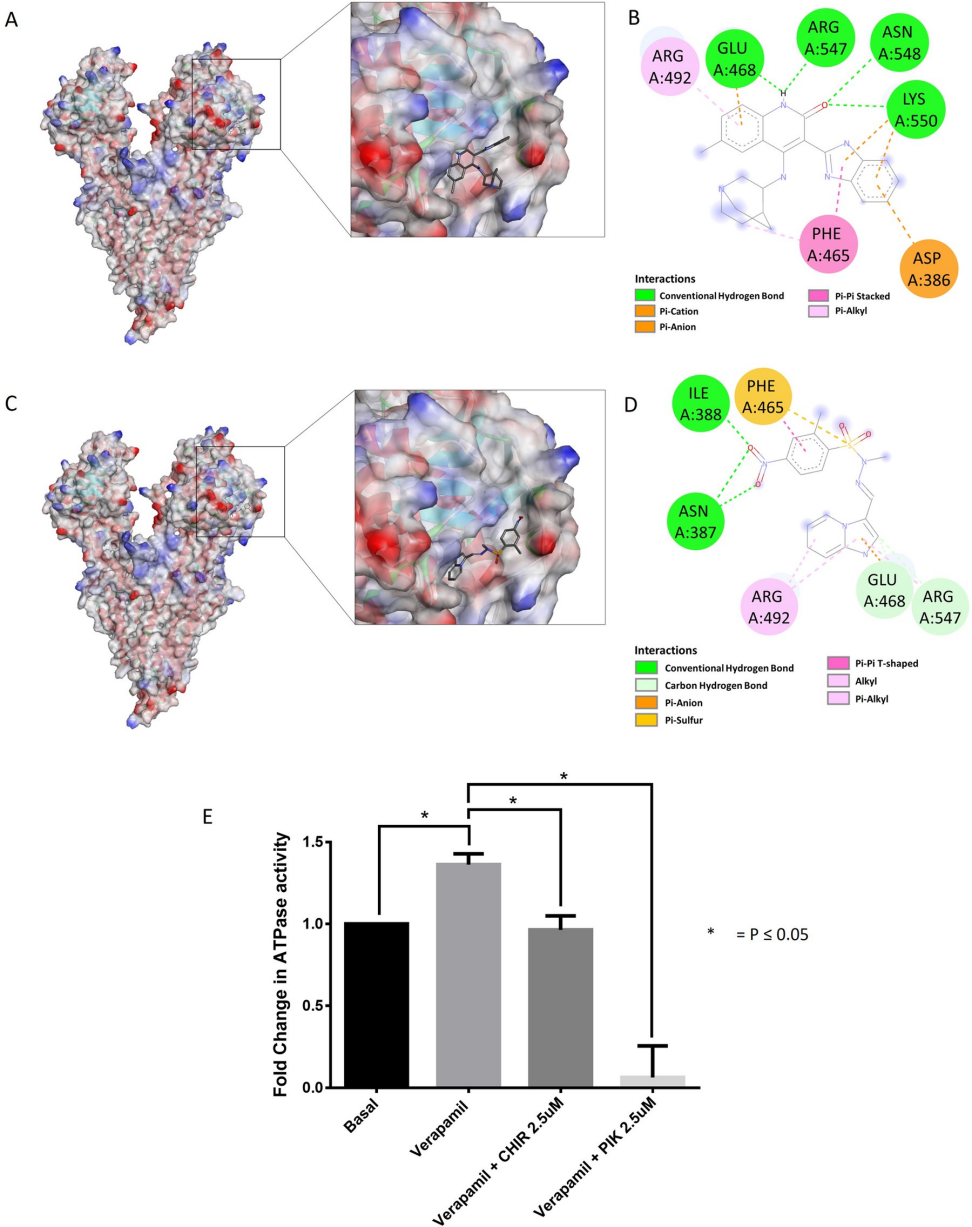
The docking of CHIR-124 and PIK-75 with Pgp. (A) Binding of CHIR-124 in the NBD1 of Pgp (PDB code: 4Q9H) (B) 2D presentation of the interactions of CHIR-124 with the amino acid residues of NBD1 (C) Binding of PIK-75 in the NBD1 of Pgp (D) 2D presentation of the interactions of PIK-75 with the amino acid residues of NBD1. (E) Inhibition of ATPase activity of Pgp by CHIR-124 and PIK-75. ATPase activity was measured using Pgp-Glo assay (Promega) as described in materials and methods. Samples were treated with 100 μM Na3VO4, 200 μM Verapamil, 2.5 μM CHIR-124 and PIK-75 or left untreated. Fold change in verapamil-treated and compound treated Pgp activity was calculated according to the manufacturer’s instructions.

After demonstrating that CHIR-124 and PIK-75 inhibited Pgp activity in the efflux assay (in both resistant cell lines) without modulating its expression, we next determined the binding of these compounds with Pgp through docking studies (see Materials and Methods). The docking suggested that the compounds bind to the NBD1 of the protein (Fig 5). Both molecules bound to the same groove in NBD1. The residues involved in the interaction included Glu 468, Arg 547, Asn 548, Lys 550 that make H-bond interactions with CHIR-124 and Ile 388, Asn 387 that make H-bond interactions with PIK-75 (Fig 5). The predictions for the interaction of CHIR-124 and PIK-75 with Pgp were supported by an *in vitro* ATPase assay for Pgp. As shown in Fig 5C, both compounds significantly inhibited verapamil-stimulated ATPase activity of Pgp, thereby validating the docking predictions.

## Discussion

Overexpression of ABC transporters confers resistance to a wide range of structurally distinct chemotherapeutic drugs and is one of the major reasons for suboptimal clinical responses in cancer treatment. Co-administration of inhibitors targeting ABC transporters have long been sought as an approach to enhance the efficacy of chemotherapeutic drugs in multidrug-resistant cancers. Three generations of ABC transporter inhibitors, however, have been unsuccessful in clinical trials due to several reasons, including adverse side effects and drug-drug interactions[41].

In the present study, we screened 1,127 inhibitors with known therapeutic targets to identify compounds with the ability to inhibit the growth of parental and multidrug-resistant cells with equal potency and thereby overcome Pgp mediated multidrug resistance. Primary and secondary screening resulted in the identification of fifteen compounds that equally inhibited the growth of both parental and resistant cells in the two cell line pairs (KB-3-1/KB-V1 and A2780/A2780-Pac-Res) at 1μM concentration. However, only four out of fifteen compounds (CHIR-124, tyrphostin-9, brefeldin-A and elesclomol) had resistant index (RI) values close to or less than 1 in both pairs of parental and resistant cell lines (Table 1). Three compounds (NVP-BVU972, zinc pyrithione and AUY922) exhibited RI of ~1 or less in A2780/A2780-Pac-Res cell line pair and RI of >2 in KB-3-1/KB-V1 pair likely due to higher expression of Pgp in KB-V1 cell line compared to A2780-Pac-Res. Another inhibitor, PIK-75, had RI of ~1 in KB-3-1/KB-V1 cell line pair while RI of >2 in the other pair which has lower expression of Pgp indicating that factors other than Pgp may be responsible for slight resistance.

Pgp-mediated multidrug resistance can be overcome by various mechanisms that include Pgp downregulation[40] inhibition of Pgp activity[10, 42] or escaping efflux through Pgp[15]. CHIR-124 equally inhibited the growth of both pairs of parental and resistant cell lines and was determined to be a Pgp inhibitor in both resistant cell lines using cell-based assays with calcein AM and rhodamine-123, two Pgp substrates. PIK-75 also inhibited the Pgp-mediated efflux of calcein AM in both the resistant cell lines despite having RI of 2.93 in A2780/A2780-Pac-Res cell line pair (S3 Fig). The Pgp inhibition was also supported by docking studies that predicted the binding of both CHIR-124 and PIK-75 to the nucleotide-binding domain (NBD) of Pgp and perturb its functional efficacy (Fig 5). Many compounds that interact with the nucleotide-binding domain (NBD) of Pgp have previously been reported to overcome multidrug resistance as they interfere with ATP hydrolysis at NBD that is essential for mediating drug transport out of the cells[25, 43]. Furthermore, an *in vitro* ATPase assay confirmed that both compounds directly inhibited the ATPase activity of Pgp.

The levels of Pgp expression have been reported to strongly correlate with the degree of drug resistance in multidrug-resistant cancer cells[44–46]. Two compounds in our screen; zinc pyrithione and AUY922, inhibited Pgp activity only in one pair of ovarian cancer cells (A2780/A2780-Pac-Res) as determined by increased calcein retention (S4 Fig). The RI values for both the inhibitors were also close to 1 in these cells. None of the inhibitors, however, inhibits Pgp activity in KB-V1 cells, where the RI for both of them was found to be more than 3 (3.02 and 4.30 for zinc pyrithione and AUY922, respectively) suggesting that higher levels of Pgp expression in these cervical cancer cells may be responsible for this resistance (Fig 1A). In agreement with our findings, AUY922 has been shown to inhibit the growth of Pgp expressing MDR cells established by continuous exposure to 17-DMAG[47]. AUY922 was shown to modulate its biomarkers in both parental and resistant cells but the mechanism through which it overcame MDR was not described. Similarly, the sensitivity of resistant cells to AUY922 was not affected by a change in the Pgp levels due to its overexpression or knockdown[47], unlike our results where KB-V1 cells expressing higher levels of Pgp are moderately resistant to AUY922, which is not able to inhibit Pgp mediated efflux in these cells.

Apart from Pgp inhibition, several drugs may bypass Pgp efflux by avoiding physical interaction with Pgp protein. Three inhibitors in our screen; elesclomol, brefeldin A and tyrphostin-9 are included in this category as they fail to increase calcein retention in KB-V1 resistant cells, despite killing both pairs of parental and resistant cells with equal GI_50_s (Table 1). This is in accordance with the finding of a previous study showing that elesclomol inhibited the growth of parental and efflux transporter overexpressing cells with equal potency in four pairs of resistant and sensitive cell lines including two that expressed Pgp (MDCK, MDCK/MDR and KB-3-1, KB-C2)[48]. How elesclomol managed to avoid efflux in these resistant cells, however, was not reported. We here rule out the inhibition of Pgp activity or downregulation of Pgp expression as mechanisms through which elesclomol overcomes MDR and propose that it avoids efflux by not being a Pgp substrate. Similarly, brefeldin A has been shown to overcome multidrug resistance and sensitize the Pgp overexpressing cells (Lovo/Dx and WIPR) against chemotherapeutic drugs by inhibiting the N-glycosylation of the Pgp as shown by gel mobility shift in a western blot[49]. However, we did not find any change in the gel mobility of Pgp from cells treated with brefeldin A. NVP-BVU972, a c-MET inhibitor, also overcomes MDR in A2780-Pac-Res cells without inhibiting Pgp (S5 Fig). Although cells expressing MDR1 have been shown to be differentially sensitive to c-MET downregulation by RNAi[50], A2780 cells are not known to express c-MET[51]. Several tyrosine kinase inhibitors (TKIs) can interact and inhibit efflux transporters MDR1 and MRP1[52], however, tyrphostin-9, a PDGFR inhibitor identified in this screen did not inhibit Pgp in cells.

Identification of drugs that inhibit Pgp as a secondary target (in addition to their primary cellular targets) or avoid efflux through it can have advantages over Pgp inhibitors that sensitize cells to various chemotherapeutic or targeted drugs. A drug able to inhibit critical cellular target(s) in the presence of Pgp overexpression, for example, can avoid challenges associated with the co-administration of the two or more drugs such as additive toxicities and differing pharmacokinetic properties[13, 53]. We, therefore, focused our efforts on the inhibitors that potently reduced cell proliferation (>50% growth inhibition at 1 μM concentrations); There were, however, twenty six other inhibitors identified in our primary screen with growth inhibition of >35% in either cell line (with <10% difference of growth inhibition in both cell lines) that we did not explore (S2 Table). Further characterization of these inhibitors can result in the identification of more compounds with the ability to overcome MDR in Pgp overexpressing cells. Taken together, our results identify inhibitors with various cellular targets that can overcome MDR in Pgp overexpressing cells. We show that some of these inhibitors can inhibit Pgp activity in cells while others avoid efflux by not being Pgp substrates. The chemotypes identified in this study can be further explored for more potent compounds for Pgp inhibition or evasion of efflux. Additional studies are needed to evaluate *in vivo* efficacy of these inhibitors in Pgp expressing xenograft models and their ability to inhibit other efflux pumps.

## Supporting information

S1 Fig Compounds which were significantly more potent in the parental KB-3-1 cell line as compared to the multidrug-resistant KB-V1 cell line. Percentage growth inhibition at 1μM concentrations of different compounds in a 3-day SRB proliferation assay is shown.
(TIF)

S2 Fig Rhodamine 123 efflux assay in A2780 and A2780-Pac-Res cell lines. Rhodamine 123 retention in parental and resistant cell lines following treatment with DMSO, verapamil (40μM) and CHIR-124 (10μM).
(TIF)

S3 Fig Pgp inhibition by PIK-75 in KB-V1 and A2780-Pac-Res cell lines. Calcein retention in KB-V1 cells following treatment with 2.5 μM and 5 μM PIK-75 analyzed through FACS (*A*) or immunofluorescence (*B*). *C*. Calcein retention in A2780-Pac-Res cells following treatment with 2.5 μM and 5 μM PIK-75 analyzed through FACS.
(TIF)

S4 Fig Pgp inhibition by AUY922 and zinc pyrithione MDR cell lines. Calcein retention through FACS analysis in A2780/A2780-Pac-Res (*A*) and KB-3-1/KB-V1 (*B*) cells treated DMSO, verapamil (40 μM), AUY922 (1.25 μM and 2.5 μM) and zinc pyrithione (1.25 μM and 2.5 μM).
(TIF)

S5 Fig Pgp Efflux assay with NVP-BVU972. Calcein AM efflux assay with the indicated concentrations of NVP-BVU972 in A2780-Pac-Res cell line.
(TIF)

S6 Fig Pgp expression level after inhibitor treatment. A2780-Pac-Res cells were treated with the indicated concentrations of different inhibitors for 24 hours, and Pgp expression was analysed through western blotting. Alpha-tubulin was used as a loading control.
(TIF)

S1 Table Percentage inhibition of cell viability for primary screening in KB-3-1 and KB-V1 cells lines. Library of 1127 compounds was screened in KB-3-1 and KB-V1 cell lines at 1μM concentration using the 3-day SRB assay. Percentage inhibition was calculated by normalizing values to DMSO control. The difference in inhibition of KB-3-1 and KB-V1 cell viability was calculated by subtracting percentage inhibition of KB-V1 from percentage inhibition of KB-3-1.
(XLSX)

S2 Table Inhibitors identified in the primary screen growth inhibition of >35% in either cell line and the difference in growth inhibition of <10% between the two cell lines. (XLSX)
