# Supplementary figures and images for: A cell-based high-throughput screen identifies inhibitors that overcome P-glycoprotein (Pgp)-mediated multidrug resistance
Source: PLoS One. 2020 Jun 2;15(6):e0233993. doi: 10.1371/journal.pone.0233993 (PMC7266297; doi:10.1371/journal.pone.0233993)

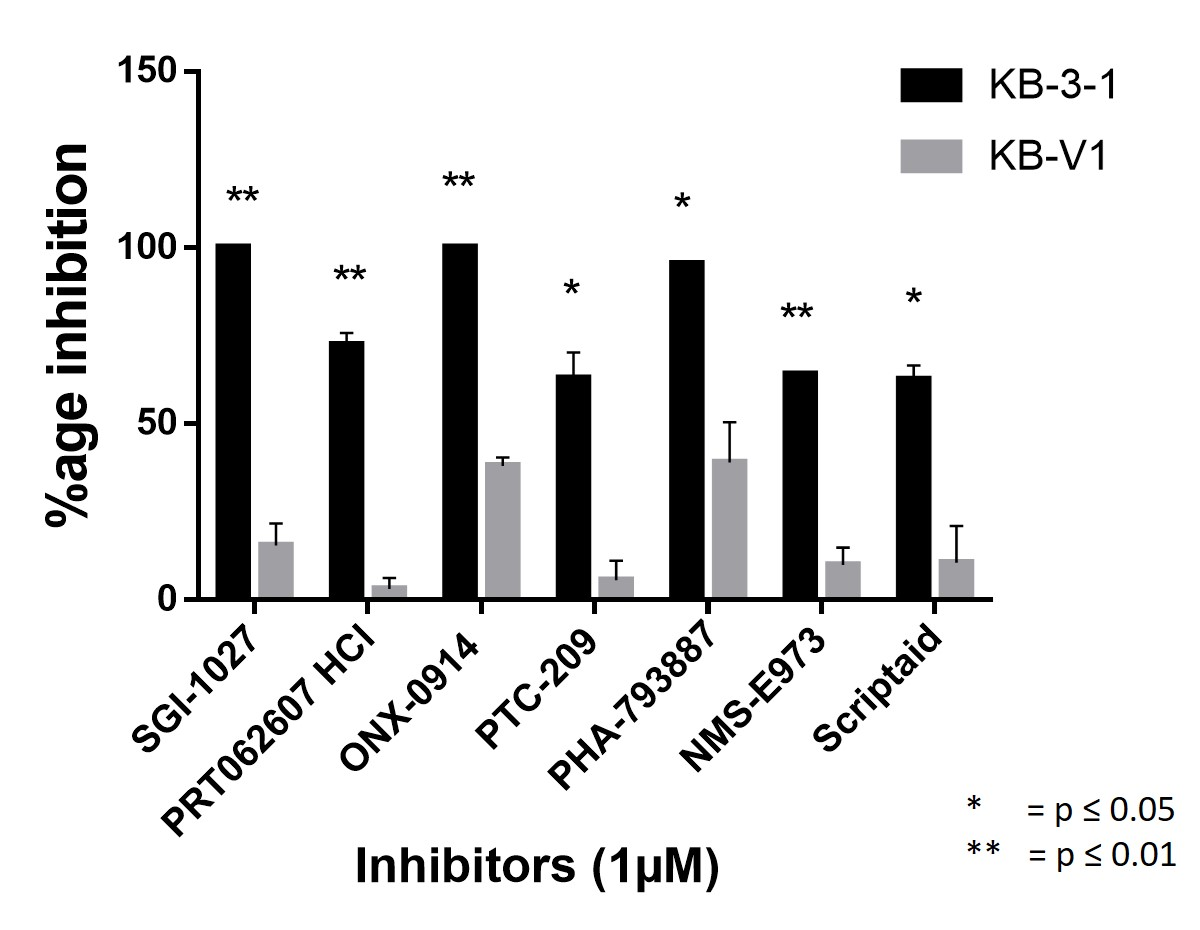

Supplement: S1 Fig — Percentage growth inhibition at 1μM concentrations of different compounds in a 3-day SRB proliferation assay is shown. (TIF) [file pone.0233993.s001.tif]

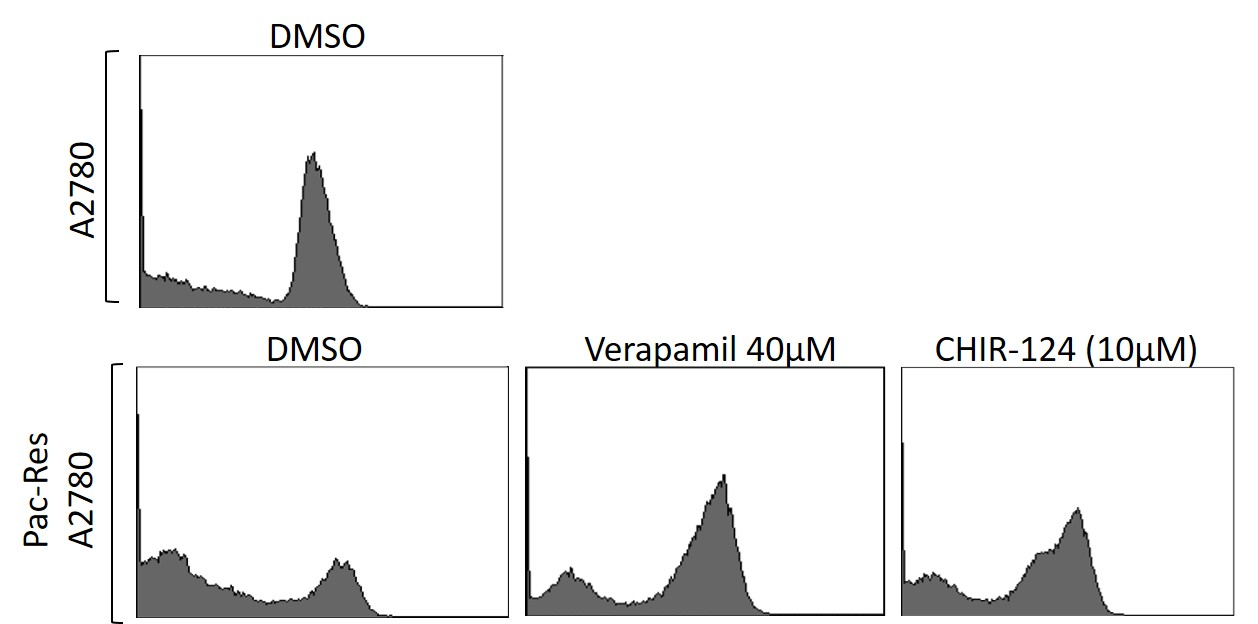

Supplement: S2 Fig — Rhodamine 123 retention in parental and resistant cell lines following treatment with DMSO, verapamil (40μM) and CHIR-124 (10μM). (TIF) [file pone.0233993.s002.tif]

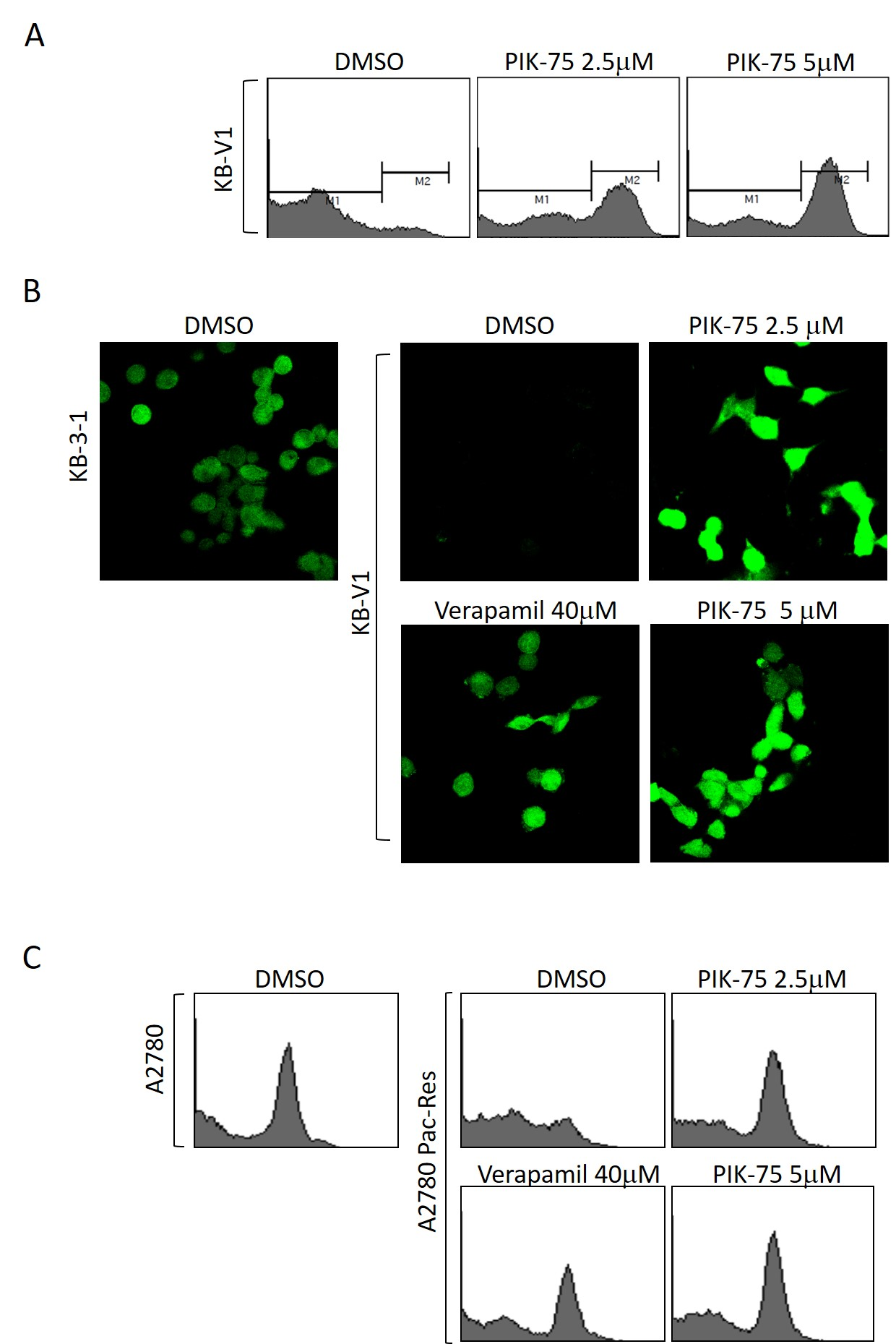

Supplement: S3 Fig — Calcein retention in KB-V1 cells following treatment with 2.5 μM and 5 μM PIK-75 analyzed through FACS (A) or immunofluorescence (B). C. Calcein retention in A2780-Pac-Res cells following treatment with 2.5 μM and 5 μM PIK-75 analyzed through FACS. (TIF) [file pone.0233993.s003.tif]

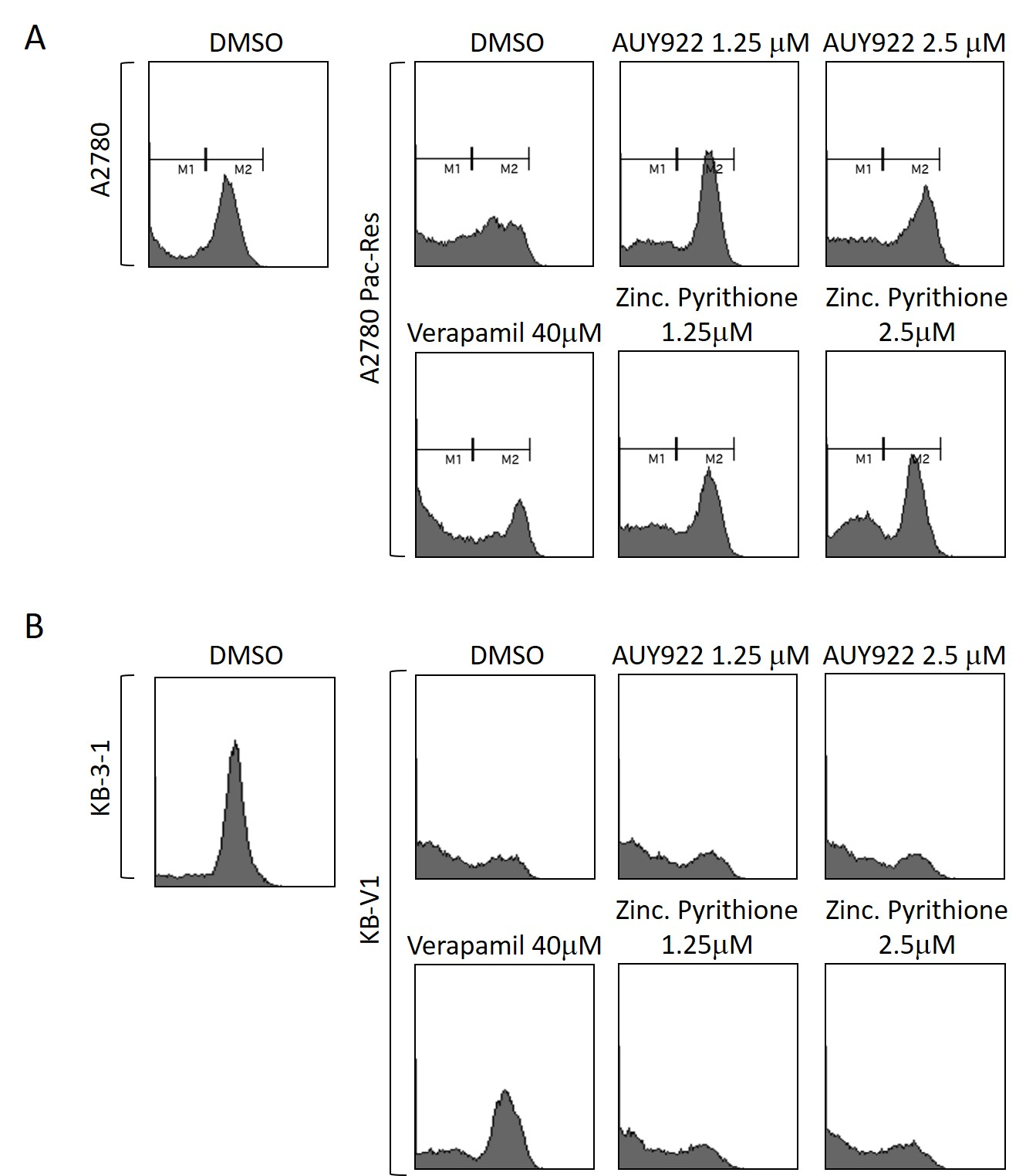

Supplement: S4 Fig — Calcein retention through FACS analysis in A2780/A2780-Pac-Res (A) and KB-3-1/KB-V1 (B) cells treated DMSO, verapamil (40 μM), AUY922 (1.25 μM and 2.5 μM) and zinc pyrithione (1.25 μM and 2.5 μM). (TIF) [file pone.0233993.s004.tif]

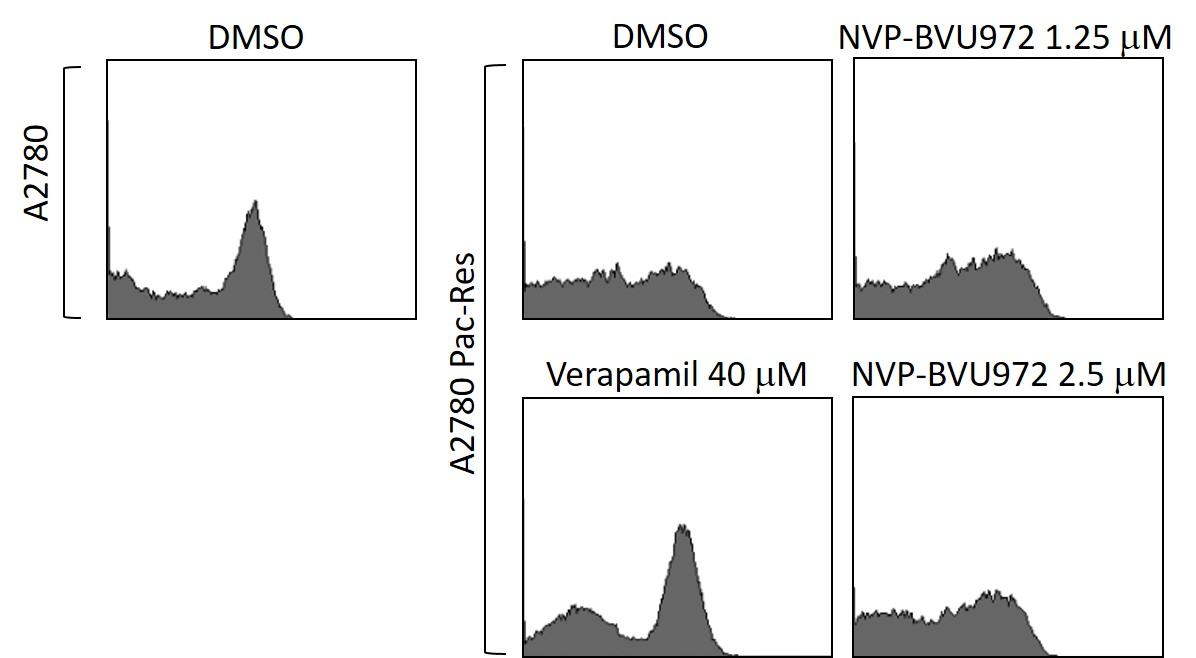

Supplement: S5 Fig — Calcein AM efflux assay with the indicated concentrations of NVP-BVU972 in A2780-Pac-Res cell line. (TIF) [file pone.0233993.s005.tif]

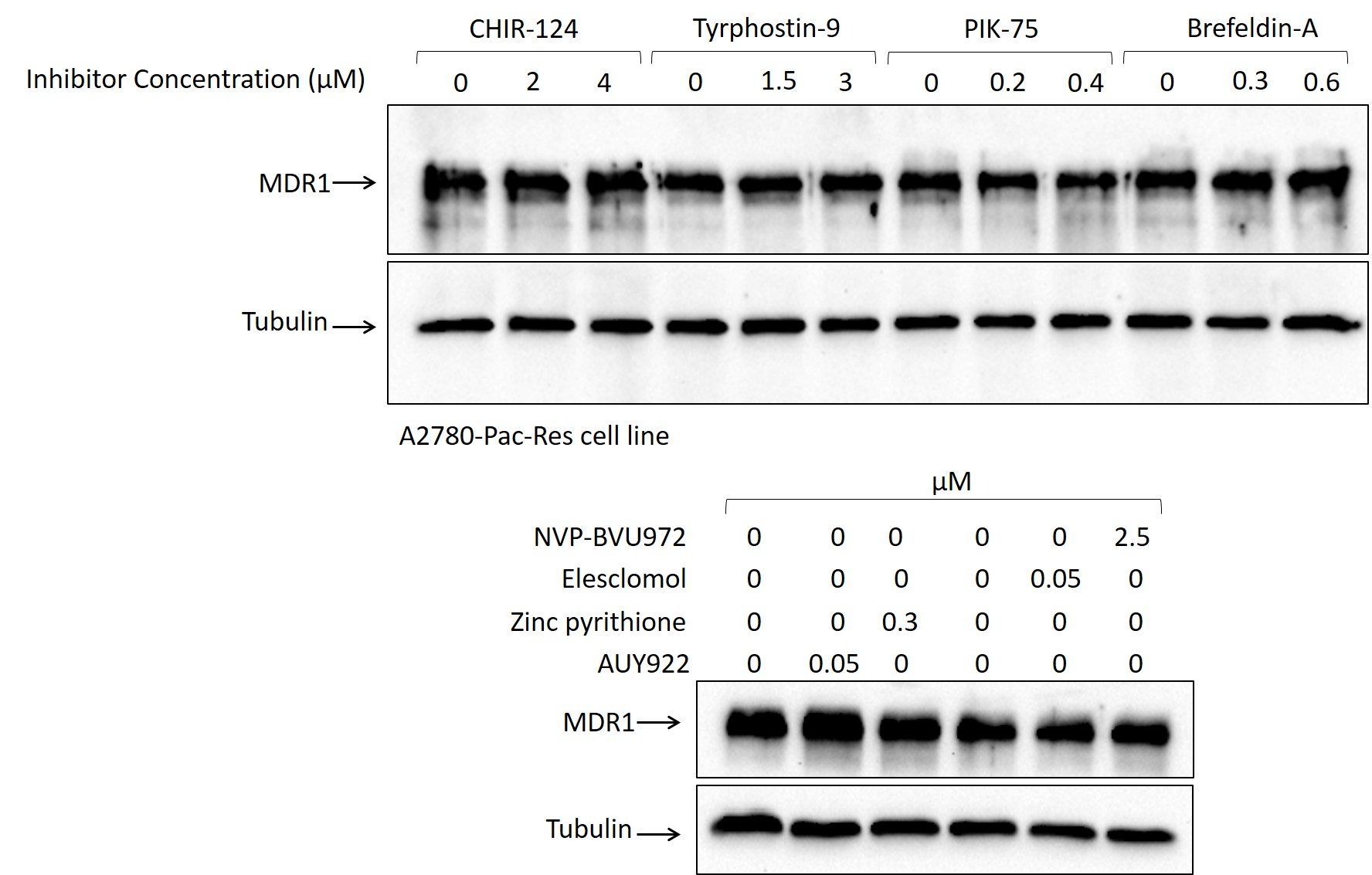

Supplement: S6 Fig — A2780-Pac-Res cells were treated with the indicated concentrations of different inhibitors for 24 hours, and Pgp expression was analysed through western blotting. Alpha-tubulin was used as a loading control. (TIF) [file pone.0233993.s006.tif]
